# Surface modification of layered perovskite Sr_2_TiO_4_ for improved CO_2_ photoreduction with H_2_O to CH_4_

**DOI:** 10.1038/s41598-017-16605-w

**Published:** 2017-11-27

**Authors:** Byeong Sub Kwak, Jeong Yeon Do, No-Kuk Park, Misook Kang

**Affiliations:** 1 Department of Chemistry, College of Natural Sciences, Yeungnam University, Gyeongsan, Gyeongbuk, 38541 Republic of Korea; 2 School of Chemical Engineering, Yeungnam University, Gyeongsan, Gyeongbuk, 38541 Republic of Korea

## Abstract

Layered perovskite Sr_2_TiO_4_ photocatalyst was synthesized by using sol-gel method with citric acid. In order to increase the surface area of layered perovskite Sr_2_TiO_4_, and thus to improve its photocatalytic activity for CO_2_ reduction, its surface was modified via hydrogen treatment or exfoliation. The physical and chemical properties of the prepared catalysts were characterized by X-ray diffraction, high-resolution transmission electron microscopy, elemental mapping analysis, energy-dispersive X-ray spectroscopy, N_2_ adsorption-desorption, UV-Vis spectroscopy, X-ray photoelectron spectroscopy, photoluminescence, and electrophoretic light scattering. CO_2_ photoreduction was performed in a closed reactor under 6 W/cm^2^ UV irradiation. The gaseous products were analyzed using a gas chromatograph equipped with flame ionization and thermal conductivity detectors. The exfoliated Sr_2_TiO_4_ catalyst (E-Sr_2_TiO_4_) exhibited a narrow band gap, a large surface area, and high dispersion. Owing to these advantageous properties, E-Sr_2_TiO_4_ photocatalyst showed an excellent catalytic performance for CO_2_ photoreduction reaction. The rate of CH_4_ production from the photoreduction of CO_2_ with H_2_O using E-Sr_2_TiO_4_ was about 3431.77 μmol/g_cat_ after 8 h.

## Introduction

As is well known, the temperature of Earth is increasing owing to greenhouse gas emissions, and CO_2_ makes up the largest portion of these greenhouse gases. The United Nations Framework Convention on Climate Change (UNFCCC) has concluded that, to maintain a stable environmental, global warming since industrialization must not exceed 2 °C. However, according to their investigation, the average ground temperature since industrialization has already increased by 0.75 °C. When we consider the heat absorbed by the sea, this temperature increases by a further 0.6 °C. Consequently, we have a temperature increase of only 0.65 °C until the temperature change set by the UNFCCC is already reached. Because of the global warming, various natural disasters have been occurred^1^. In order to avoid this problem, we must reduce CO_2_ emissions. However, it is impossible to stop CO_2_ emissions completely because of the current industrial systems are depending on the fossil fuels.

Carbon capture and storage (CCS) technology has been developed in order to reduce and/or control the CO_2_ emissions^2,3^. However, this CCS technology has several problems such as high cost, additional energy requirements, stability, and storage limitations. Therefore, carbon capture and utilization (CCU) technology is expected to replace it^4–6^, and is advantageous in economic and environmental view because it can prevent CO_2_ emissions, and convert them into useful materials. There are various strategies for CO_2_ utilization. One is the non-conversional use of CO_2_, and the others involve its conversion using chemical, biochemical, photochemical, and electrochemical methods. Of these, the photochemical method is a more promising technology in environmental terms. CO_2_ photoreduction using a photocatalyst can generate useful compounds such as CH_4_, HCOOH, HCHO, and CH_3_OH^7^. Much research has been devoted to this field since the first report by Inoue *et al*.^8^ on the photoreduction of CO_2_, and many different semiconductors have been developed and used as photocatalysts, such as TiO_2_^9^, WO_3_^10^, ZnO^11^, GaP^12^, CdS^13^, and SiC^14^. Of these, TiO_2_ is one of the most well-known and widely used materials. In order to improve the optical properties of TiO_2_, it has been combined with various metals to form hybridized composites such as Ag/TiO_2_^15^, Pt/TiO_2_^16^, Ru/TiO_2_^17^, Pd/TiO_2_^18^, Ni/TiO_2_^19^, Cu/TiO_2_^20^, TiO_2_/Cu-TiO_2_^21^, CeO_2_-TiO_2_^22^, MgO-TiO_2_^23^, NiO-In_2_O_3_/TiO_2_^24^, CuS_x_-TiO_2_^25^, NiS-TiO_2_^26^, In_2_O_3_/TiO_2_^27^, TiO_2_/Fe-TiO_2_^28^ and multi-walled carbon nanotube (MWCNT)@TiO_2_^29^ in an attempt to reduce the band gap or suppress recombination of photogenerated charge carriers. Also, catalysts having a combination of an organic and metal material such as g-C_3_N_4_^30^, Ni_12_P_5_/g-C_3_N_4_^31^, Au cluster-NP/C_3_N_4_^32^, AgX/g-C_3_N_4_ (X = Cl and Br)^33^, RuRu′/Ag/NS-C_3_N_4_^34–37^ and Co-ZIF^38–41^ are being developed.

Recently, the development of TiO_2_ photocatalysts with the perovskite structure ABO_3_, has attracted due to the unique perovskite structure, their composition can be easily changed at the A, and B sites and the metal introduced can be quantitatively substituted into the skeleton. Among the perovskite semiconductors, SrTiO_3_ is widely used as a photocatalyst. Much like TiO_2_, SrTiO_3_ has been combined with other species to form hybrid composites such as Mn/SrTiO_3_^42^, Cu/SrTiO_3_^43^, N-doped TiO_2_-SrTiO_3_^44^, Fe_2_O_3_/SrTiO_3_^45^, SrTiO_3_:Cr/Ta/F^46^, SrTiO_3_/HZSM-5^47^, SrTiO_3_/TiO_2_/H-titanate nanofiber^48^, SrTiO_3_:Rh/Sb^49^, La/Cr-doped SrTiO_3_^50^, Pt/SrTiO_3_^51^, Zn/SrTiO_3_^52^, Ag_3_PO_4_/Cr-SrTiO_3_^53^, and g-C_3_N_4_-SrTiO_3_:Rh^54^ to improve its photocatalytic performance. Studies on other perovskite catalysts, including Ca_x_Ti_y_O_3_^55^, and basalt fiber@PbTiO_3_^56^, also have been recently reported.

**Figure 1:**
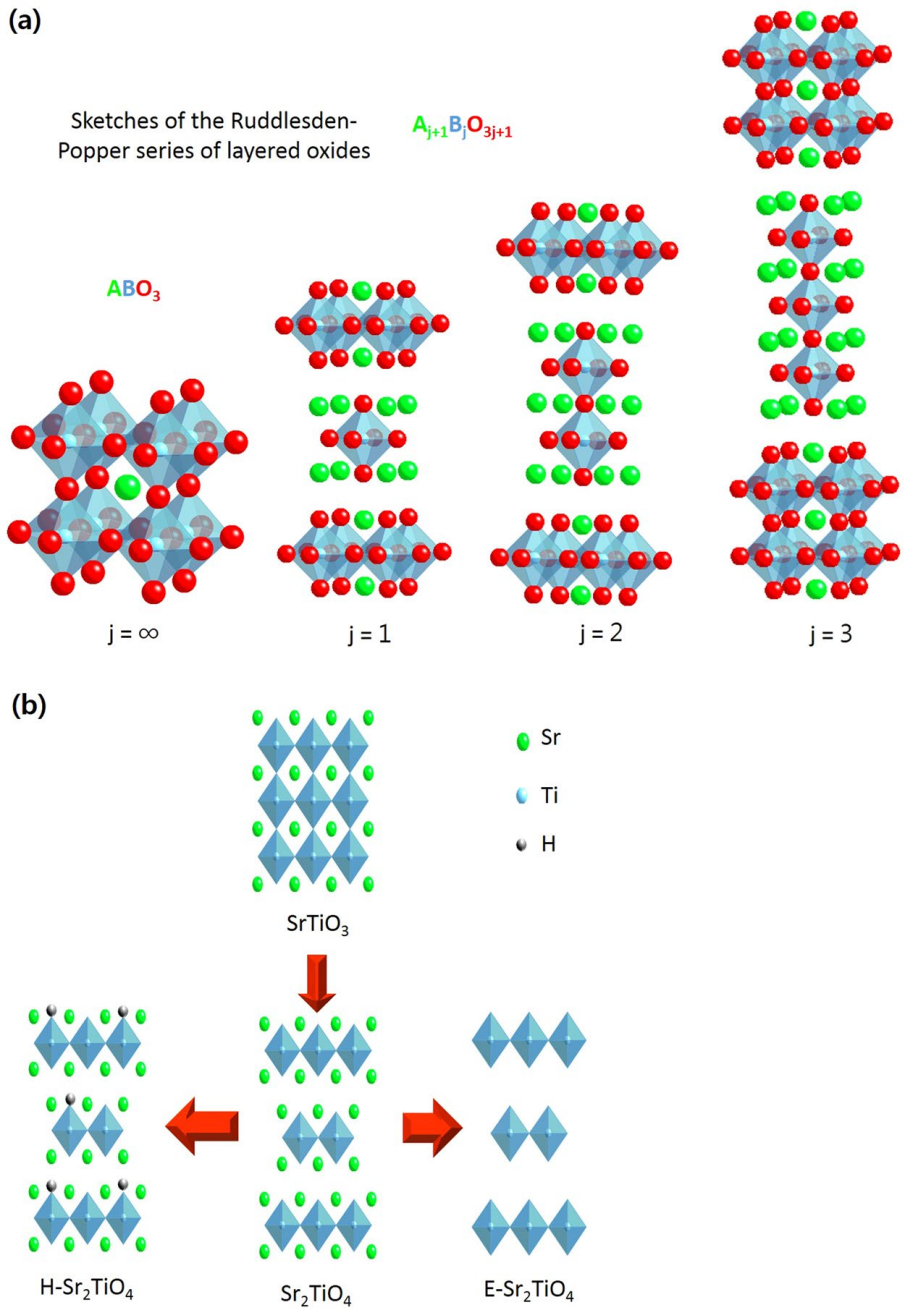
(**a**) Ruddlesden-Popper phase of layered oxide and (**b**) the overview of catalyst surface modification.

Another advantage of perovskite is that it forms a layered perovskite depending on the nature and contents of the A and B ions. Figure 1a shows the structure of a Ruddlesden-Popper A_j+1_B_j_O_3j+1_ perovskite. Here, when the j value increases, the structure tends towards an ABO_3_ perovskite. In particular, A_2_BO_4_, for which j = 1, shows a layered structure with a large gap between each BO_6_ octahedron.

In this study, we have attempted to improve the photocatalytic performance of Sr_2_TiO_4_ layered perovskite by increasing its surface area (Fig. 1b). The surface of the synthesized catalyst was hydrogen treated or exfoliated to increase its interaction with the CO_2_ feed material and thus amplify its CO_2_ photoreduction activity. The characteristic properties of the synthesized catalysts were measured by using a variety of techniques such as using X-ray diffraction (XRD), high-resolution transmission electron microscopy (HR-TEM), N_2_ adsorption-desorption isotherm analysis, UV-Vis spectroscopy, photoluminescence (PL), zeta potential analysis, and X-ray photoelectron spectroscopy (XPS). Furthermore, their photocatalytic activity for the reduction of CO_2_ with H_2_O under UV light was studied.

## Results

### X-ray diffraction (XRD) patterns

**Figure 2:**
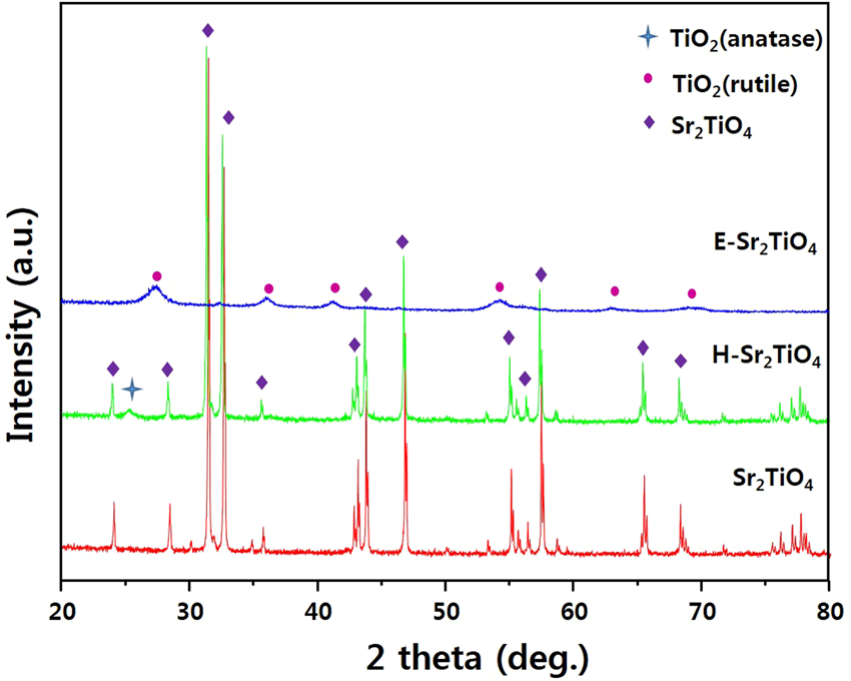
XRD patterns of Sr_2_TiO_4_, H-Sr_2_TiO_4_ and exfoliation Sr_2_TiO_4_ catalysts.

The XRD patterns for Sr_2_TiO_4_, H-Sr_2_TiO_4_, and E-Sr_2_TiO_4_ are given in Fig. 2. The peaks for Sr_2_TiO_4_ are observed at 2θ = 24.076, 28.386, 31.464, 32.656, 35.673, 42.899, 43.217, 43.852, 46.791, 55.049, 55.605, 56.319, 57.431, 65.452, and 68.390°, which are assigned to the (011), (004), (013), (110), (112), (015), (006), (114), (020), (116), (024), (017), (123), (026), and (220) planes of Sr_2_TiO_4_, respectively, identifying it as a layered perovskite-type tetragonal structure (JCPDS 00-039-1471)^57^. The crystallite size of Sr_2_TiO_4_ calculated using Scherer’s equation based on the (013) plane is 12.856 Å^58^. After hydrogen treatment, an anatase TiO_2_ peak is observed at 2θ = 25.429°. However, the other peaks for the Sr_2_TiO_4_ structure are maintained, and the crystallite sizes based on the Sr_2_TiO_4_ (013) plane and the TiO_2_ anatase (011) plane are 9.371 and 3.024 Å, respectively. Conversely, the Sr_2_TiO_4_ sample exfoliated with HNO_3_ and TPAOH presents a different XRD pattern. The peaks for E-Sr_2_TiO_4_ are found at 27.141, 36.070, 41.232, 54.414, 63.069, and 69.105°, which are assigned to the (110), (011), (111), (121), (130), and (031) planes of rutile TiO_2_, respectively^59^. These structural changes are due to the fact that the Sr ions located between the Sr_2_TiO_4_ layers are removed by HNO_3_ treatment, and only the TiO_6_ octahedra (corresponding to BO_6_) remain. The crystal structure analysis reveals that the Ti forms a rutile structure in the Sr_2_TiO_4_ and that the Sr is intercalated between the layers. The crystallite size of the catalysts was calculated using scherrer’s equation. In the case of Sr_2_TiO_4_ and H-Sr_2_TiO_4_, (013) plane was selected, and (110) plane was selected for E-Sr_2_TiO_4_. As a result, the crystallite size of Sr_2_TiO_4_, H-Sr_2_TiO_4_, and E-Sr_2_TiO_4_ were found to be 11.568, 11.578, 1.165 Å, respectively.

### High-resolution transmission electron microscopy (HR-TEM), element mapping and EDX analysis

**Figure 3:**
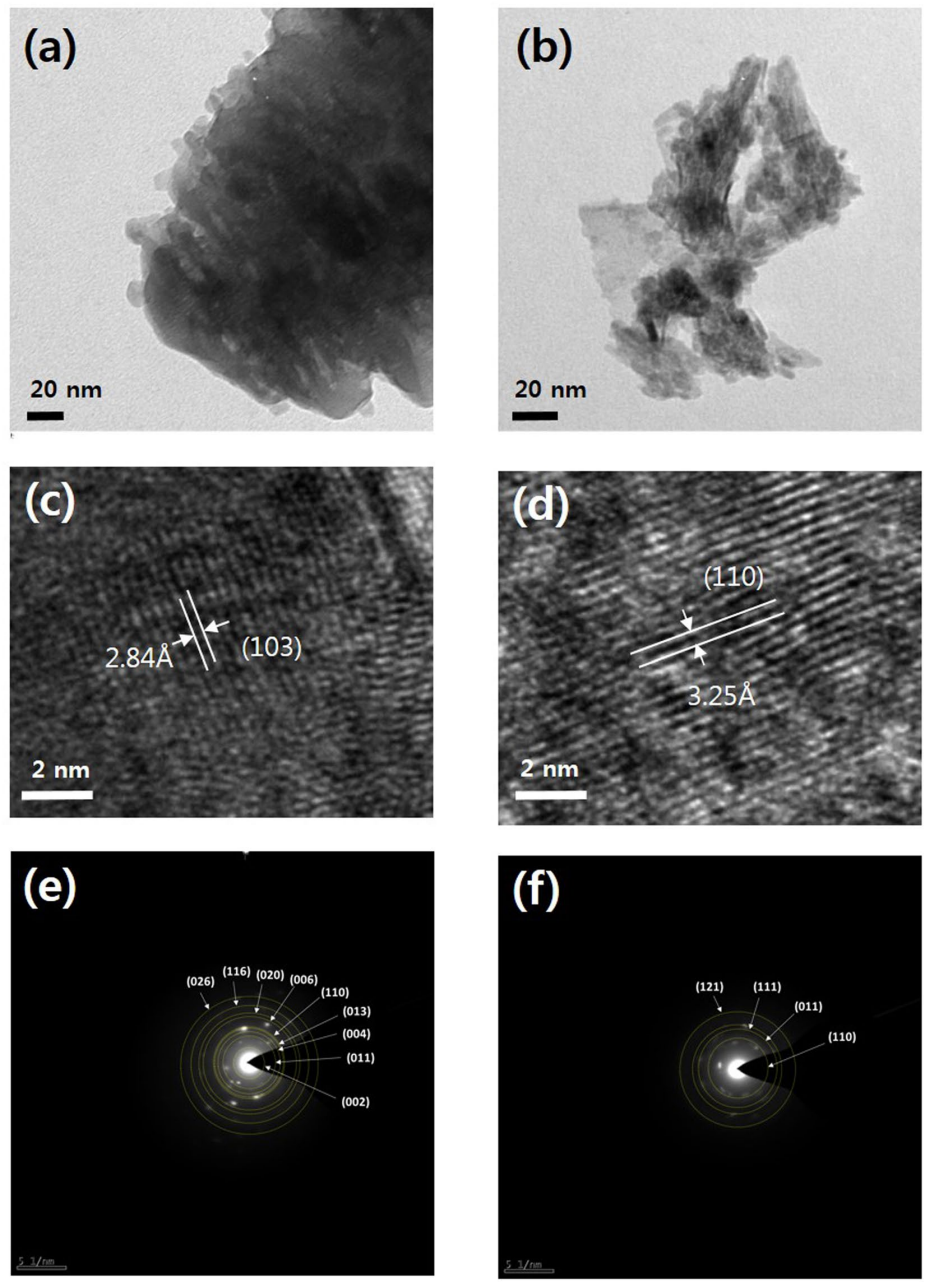
High resolution TEM and SAED images of catalysts: (**a**), (**c**) Sr_2_TiO_4_ and (**b**), (**d**) exfoliation Sr_2_TiO_4_.

The differences in the morphologies of Sr_2_TiO_4_ and E-Sr_2_TiO_4_ were investigated using HR-TEM and selected area electron diffraction (SAED) (Fig. 3). The larger particles are observed in Sr_2_TiO_4_, and whereas E-Sr_2_TiO_4_ consists of the particles form a separate sheets or randomly folded sheets. The images show that the interplanar distance for E-Sr_2_TiO_4_ is larger than that for Sr_2_TiO_4_. The d-spacings for the Sr_2_TiO_4_ (013) plane and the E-Sr_2_TiO_4_ (110) plane are 2.84 and 3.25 Å, respectively. These results are in accordance with the values derived from XRD patterns.

**Figure 4:**
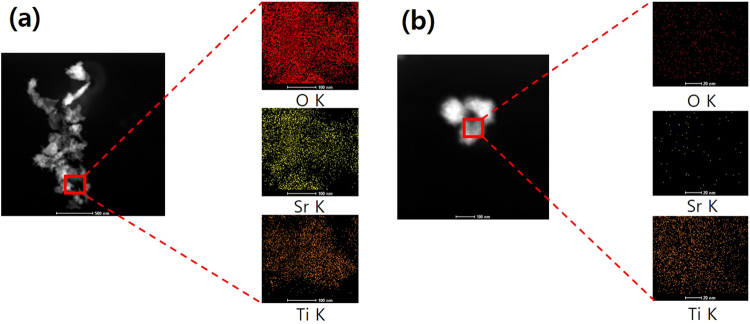
HR-TEM elemental mapping analysis of catalysts: (**a**) Sr_2_TiO_4_ and (**b**) exfoliation Sr_2_TiO_4_.

The compositions of Sr_2_TiO_4_ and E-Sr_2_TiO_4_ were analyzed by HR-TEM element mapping analysis and EDX, and the results are shown in Fig. 4 and Table 1. In Sr_2_TiO_4_, Sr and Ti ions are uniformly distributed throughout the particles and Sr ions are more abundant than Ti ions. The atomic percent values for Sr and Ti are 16 and 11%, respectively. However, the Sr ion content of E-Sr_2_TiO_4_ is much lower than that of Sr_2_TiO_4_. Furthermore, EDX analysis showed that the Sr ion content, which is about 1.4-times that of Ti in Sr_2_TiO_4_, is reduced to just 0.07% that of Ti in E-Sr_2_TiO_4_. Thus, these results support the assertion that the layers are separated because Sr ions are removed from the interlayers by exfoliation.

**Table 1:** Atomic ratio by HR-TEM EDX analysis.

Catalyst	(Atomic %)		
	Sr	Ti	O
Sr_2_TiO_4_	16.16	11.93	71.89
E-Sr_2_TiO_4_	4.80	68.29	26.90

### Brunaure-Emmett-Teller (BET) surface area analysis

**Figure 5:**
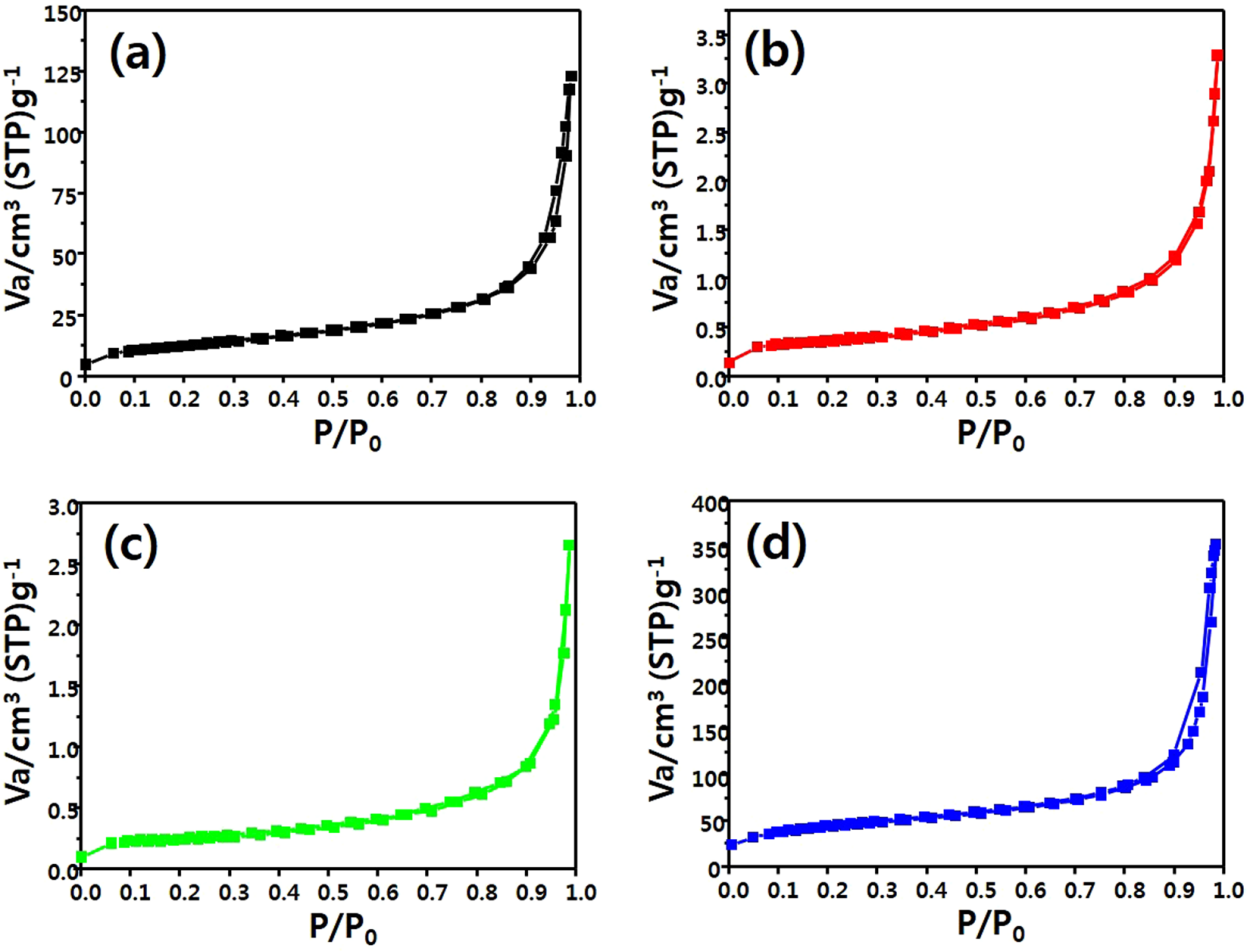
N_2_ adsorption and desorption isotherms of catalysts; (**a**) P-25, (**b**) Sr_2_TiO_4_, (**c**) H-Sr_2_TiO_4_ and (**d**) E-Sr_2_TiO_4_.

Figure 5 shows the N_2_ adsorption-desorption isotherms at 77 K for P-25, which was used as a comparative sample, Sr_2_TiO_4_, H-Sr_2_TiO_4_, and E-Sr_2_TiO_4_. According to the IUPAC classification, the adsorption-desorption isotherm curves of all the catalysts belong to type III. Therefore, the synthesized catalysts are non-porous materials. However, the slight hysteresis in the curves is due to the bulk pores between the particles. The specific surface areas of Sr_2_TiO_4_ and H-Sr_2_TiO_4_ are 1.19 and 0.78 m^2^/g, respectively, which are very low. However, E-Sr_2_TiO_4_ has a specific surface area of 358.54 m^2^/g, which is much larger than those of Sr_2_TiO_4_ and H-Sr_2_TiO_4_. The increase in the specific surface are of E-Sr_2_TiO_4_ is due not only to the removal of Sr ions from between the layers, but also to the separation of the layers, as shown in the HR-TEM images. The increase in catalyst surface area leads to an increase in the number of active sites for CO_2_ and H_2_O to react, leading to an increase in reactivity. Therefore, E-Sr_2_TiO_4_ was expected to exhibit improved catalytic activity compared to those of Sr_2_TiO_4_ and H-Sr_2_TiO_4_. This specific surface area is considerably larger than that of the commercial catalyst P-25, which is 43.65 m^2^/g.

### Optical properties of photocatalysts

**Figure 6:**
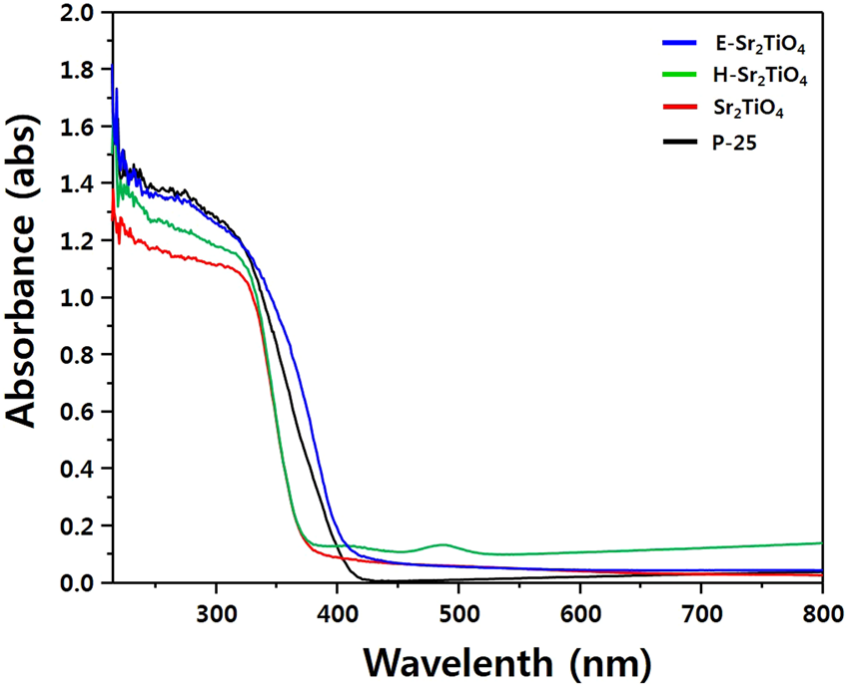
UV-visible spectra of catalysts.

**Figure 7:**
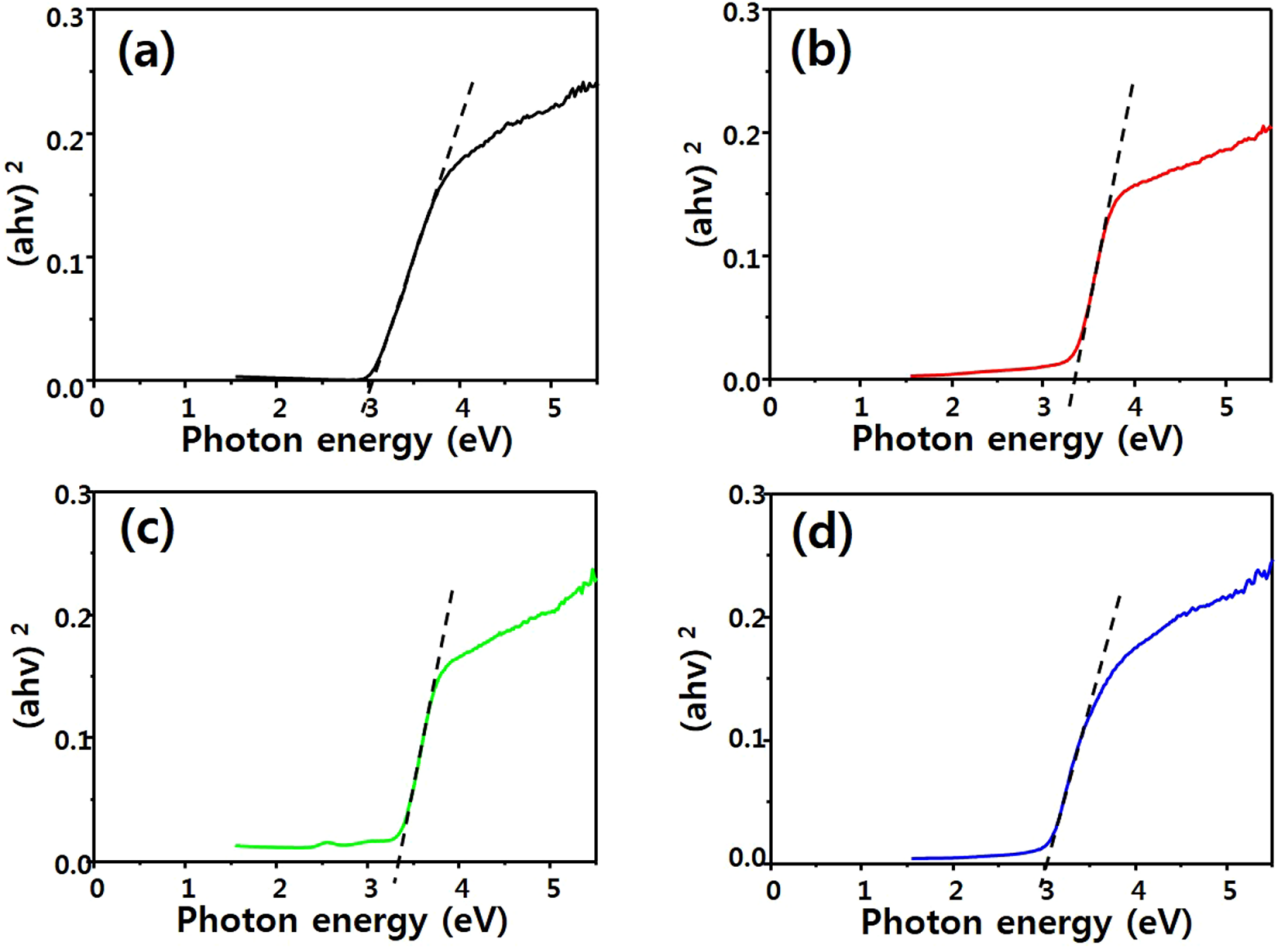
Tauc plot of catalysts; (**a**) P-25, (**b**) Sr_2_TiO_4_, (**c**) H-Sr_2_TiO_4_ and (**d**) E-Sr_2_TiO_4_.

Figures 6 and 7 show the UV-Vis spectra and Tauc’s plots of P-25, Sr_2_TiO_4_, H-Sr_2_TiO_4_, and E-Sr_2_TiO_4_. The UV absorptions of Sr_2_TiO_4_ and H-Sr_2_TiO_4_ are blue-shifted compared to that of P-25 because of the influence of SrO, with its band gap of 5.71 eV^60^. Conversely, the absorbance of E-Sr_2_TiO_4_ is shifted to a longer wavelength due to the removal of Sr ions. Most interestingly, it moved to longer wavelength than that of P-25. This is because E-Sr_2_TiO_4_ has a rutile TiO_2_ structure (band gap: 3.0 eV), as confirmed by the XRD analysis above. Therefore, the absorbance of E-Sr_2_TiO_4_ is shifted to longer wavelength than that of P-25, which composed mainly of anatase TiO_2_ (band gap: 3.2 eV). The band gap was calculated using the Tauc equation^61^:

1 $${\rm{\alpha }}{\rm{h}}{\rm{\nu }}={\rm{A}}{({\rm{h}}{\rm{\nu }}-{{\rm{E}}}_{{\rm{bg}}})}^{1/2}$$

where *α*, *h*, *ν*, *A*, and *E*_bg_ represent the absorption coefficient, Plank’s constant, light frequency, a constant, and band gap energy, respectively. In a plot of (*αhν*)^2^ versus photon energy (*hν*), the intercept on the x axis gives the band gap. Using this method, the band gap of P-25, Sr_2_TiO_4_, H-Sr_2_TiO_4_ and E-Sr_2_TiO_4_ were calculated to be 3.16, 3.33, 3.34, and 3.03 eV, respectively. Therefore, E-Sr_2_TiO_4_ has the narrowest band gap, making it most suitable as a photocatalyst.

**Figure 8:**
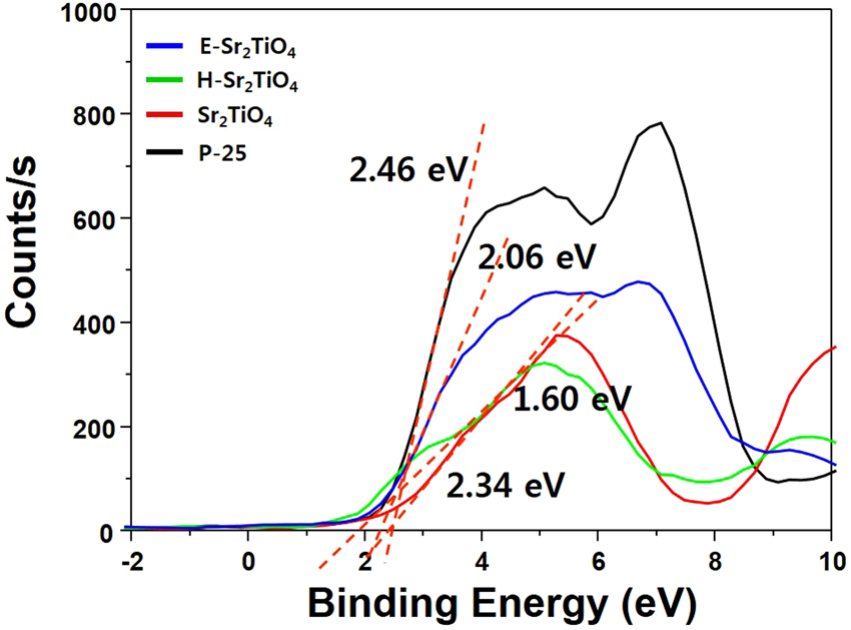
XPS valance band spectra of catalysts.

**Figure 9:**
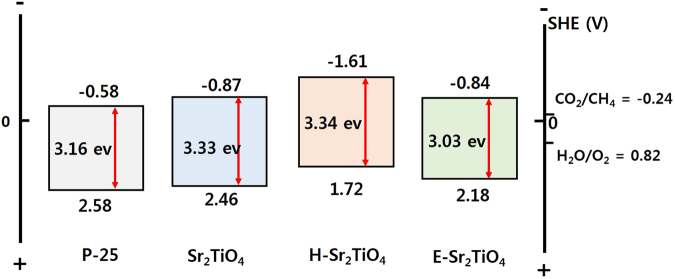
Energy diagram of catalysts.

Photocatalysts with narrower band gaps have advantages in terms of photosensitization. However, in order to exhibit good performance in the current system, the band gap of a photocatalyst should also include the CO_2_/CH_4_ and H_2_O/O_2_ reduction potentials. Figure 8 shows the XPS valance band spectra of the catalysts. Based on the data obtained, the valance band value of the catalysts was confirmed, and the values for P-25, Sr_2_TiO_4_, H-Sr_2_TiO_4_, and E-Sr_2_TiO_4_ are 2.46, 2.34, 1.60, and 2.06 eV, respectively. When the vacuum level of 4.5 eV is corrected to 0 V for a standard hydrogen electrode (SHE) and the work function of the XPS instrument is taken as 4.62 eV^62^, the valance band maximum values for P-25, Sr_2_TiO_4_, H-Sr_2_TiO_4_ and E-Sr_2_TiO_4_ are 2.58, 2.46, 1.72 and 2.18 eV (vs. SHE), respectively. According to these valance band and band gap values, the conduction band minimum values for P-25, Sr_2_TiO_4_, H-Sr_2_TiO_4_, and E-Sr_2_TiO_4_ are -0.58, 0.87, -1.61, and -0.84 eV (vs. SHE), respectively. Figure 9 shows the energy diagrams obtained for the catalysts using the valance and conduction band values and the band gap. All catalysts contain the CO_2_/CH_4_ and H_2_O/O_2_ reduction potential. Therefore, the synthesized catalysts are suitable for the photoreduction of CO_2_ with H_2_O to CH_4_.

**Figure 10:**
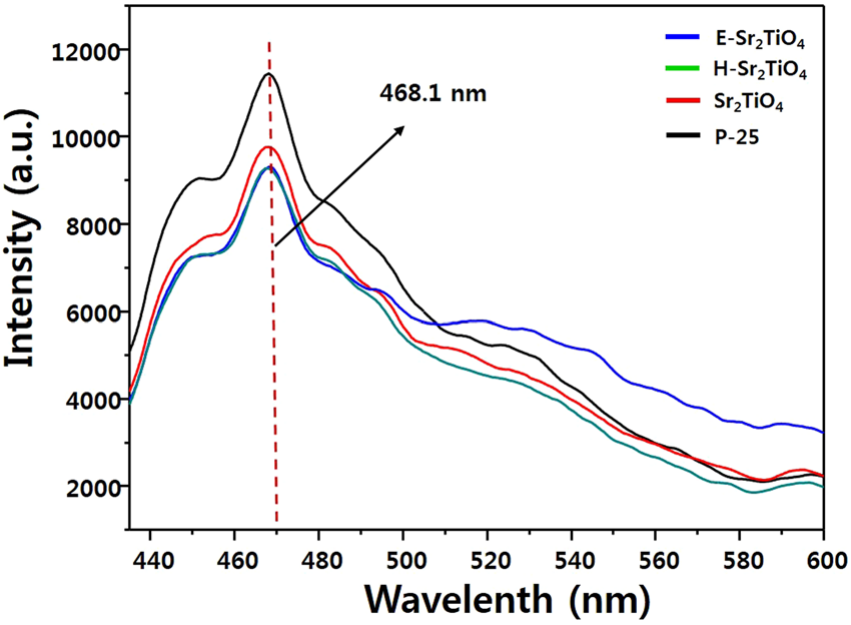
PL emission spectra of catalysts.

In order to understand the recombination of excited electrons and holes, PL analysis was conducted, and the results are shown in Fig. 10. The PL spectra of the catalysts show a strong emission signal at 468.1 nm. The PL intensity of Sr_2_TiO_4_ is smaller than that of P-25. This is due to a decrease in the number of excited electrons because of the wide band gap of Sr_2_TiO_4_. The intensity for H-Sr_2_TiO_4_ is lower than that of Sr_2_TiO_4_. This is because the oxidation state of the exposed Ti on the surface is reduced to (4-δ)^+^, which is not +4, and the reduced Ti suppresses the recombination of electrons and holes by trapping the excited electrons in the conduction band. The E-Sr_2_TiO_4_ also exhibits a PL intensity lower than that of Sr_2_TiO_4_ and much lower than that of P-25. Generally, excited electrons and holes move from the bulk of a particle to its surface where they react with reactants. The recombination of excited electrons and holes takes place in the bulk or on the surface of a particle during transport. When the particles are exfoliated, the internal area of the particles decreases and the distance to the surface for the electrons and holes decreases. Therefore, recombination inside the particles is also reduced. This is the reason that E-Sr_2_TiO_4_ has a lower PL intensity than that of P-25. Thus, the above analysis indicates that H-Sr_2_TiO_4_ and E-Sr_2_TiO_4_ will be better photocatalysts than Sr_2_TiO_4_.

### X-ray photoelectron spectroscopy (XPS) analysis

**Figure 11:**
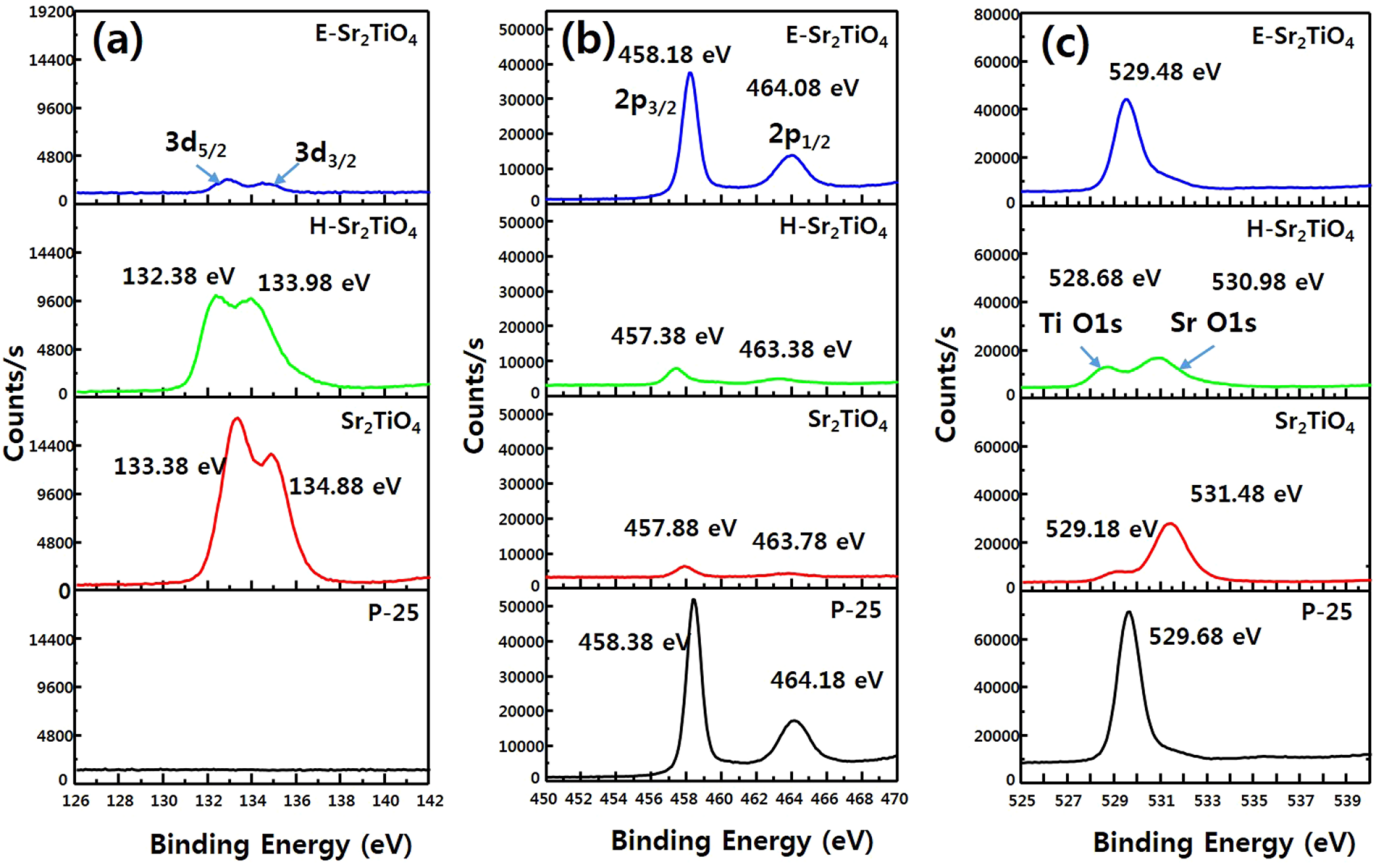
XPS spectra of catalysts; (**a**) Sr 3d spectra, (**b**) Ti 2p spectra, and (**c**) O 1s spectra.

The XPS spectra of the photocatalysts were obtained to confirm the oxidation state of the elements according to their chemical bonding, and the results are shown in Fig. 11. For Sr_2_TiO_4_, the peaks located at 133.38 and 134.88 eV are assigned to Sr-3d_5/2_ and Sr-3d_3/2_ core levels respectively. In H-Sr_2_TiO_4_, the Sr 3d peaks are shifted toward a slightly lower binding energy. In E-Sr_2_TiO_4_, the intensity of the 3d peaks is greatly reduced. This is due to the removal of Sr ions from the interlayer spaces, as described above. The Ti 2p_3/2_ and 2p_1/2_ peaks of Sr_2_TiO_4_ are observed at 457.88 and 463.78 eV, respectively. The Ti 2p peaks for H-Sr_2_TiO_4_ are shifted to a lower binding energy, similarly to the Sr 3d peaks. This is because some of the Sr^2+^ and Ti^4+^ ions are reduced by hydrogen to Sr^(2−δ)+^ and Ti^(4−δ)+^, respectively. The Ti 2p peaks for E-Sr_2_TiO_4_ have significantly different peak intensities to Sr_2_TiO_4_ or H-Sr_2_TiO_4_, and its Ti 2p_3/2_ and Ti 2p_1/2_ peaks are observed at 458.18 and 464.08 eV, respectively. This binding energy is shifted slightly lower compared to that of P-25. Therefore, the Ti ions of E-Sr_2_TiO_4_ are slightly more reduced ions than in P-25. It is believed that this can induce vacancies in the crystal framework and facilitate the movement of electrons and holes, which can be advantageous for photocatalytic activity.

There are two O 1 s peaks in Sr_2_TiO_4_ and H-Sr_2_TiO_4_. The peak at ~529 eV is from oxygen bound to Ti, and the peak at ~531 eV corresponds to oxygen bound to Sr. For E-Sr_2_TiO_4_, only the peak corresponding to oxygen bonded to Ti is observed (at 529.48 eV), because the Sr is removed by exfoliation.

### Zeta potential analysis of photocatalysts

Since the catalytic reaction takes place in H_2_O, it is important to study the dispersion of the catalyst particles in H_2_O. The zeta potentials were measured after dispersing the catalysts in distilled water or in bubbling-CO_2_ solution (i.e., the reaction conditions), and the results are shown in Table 2. Generally, a larger absolute value for the measured zeta potential means that the particles are well dispersed in a solution. The zeta potentials for Sr_2_TiO_4_, H-Sr_2_TiO_4_, E-Sr_2_TiO_4_, and P-25 are -11.99, -11.15, -42.39, and -15.61 mV, respectively. Therefore, the degree of colloidal dispersion in H_2_O follows the order E-Sr_2_TiO_4_ > P-25 > Sr_2_TiO_4_ > H-Sr_2_TiO_4_. Thus, all catalysts can be adequately dispersed in H_2_O. However, the zeta potential shows a different pattern after CO_2_ bubbling: all the negative potential values are changed to positive values. The zeta potentials for Sr_2_TiO_4_, H-Sr_2_TiO_4_, E-Sr_2_TiO_4_, and P-25 are 1.16, 16.29, 26.22, and 22.00 mV, respectively. Therefore, the degree of colloidal dispersion in the solution after CO_2_ bubbling follows the order E-Sr_2_TiO_4_ > P-25 > H-Sr_2_TiO_4_ > Sr_2_TiO_4_. Thus, E-Sr_2_TiO_4_, P-25, and H-Sr_2_TiO_4_ show good dispersion under the reaction conditions, which is considered advantageous for CO_2_ photoreduction performance. However, in the case of Sr_2_TiO_4_, the zeta potential is low, so it is likely to exhibit poor catalytic performance owing to it being more agglomerated than the other catalysts.

**Table 2:** Summary of physical and chemical properties of catalysts.

		P-25	Sr_2_TiO_4_	H-Sr_2_TiO_4_	E-Sr_2_TiO_4_
Band gap (eV)		3.16	3.33	3.34	3.03
S_BET_ (m^2^/g)		43.65	1.19	0.78	358.54
Zeta potential (mV)	Before-CO_2_	−15.61	−11.99	−11.15	−42.39
	After-CO_2_	22.00	1.16	16.29	26.22

### Photocatalytic reduction of CO_2_ with H_2_O, property after reaction, and mechanism

**Figure 12:**
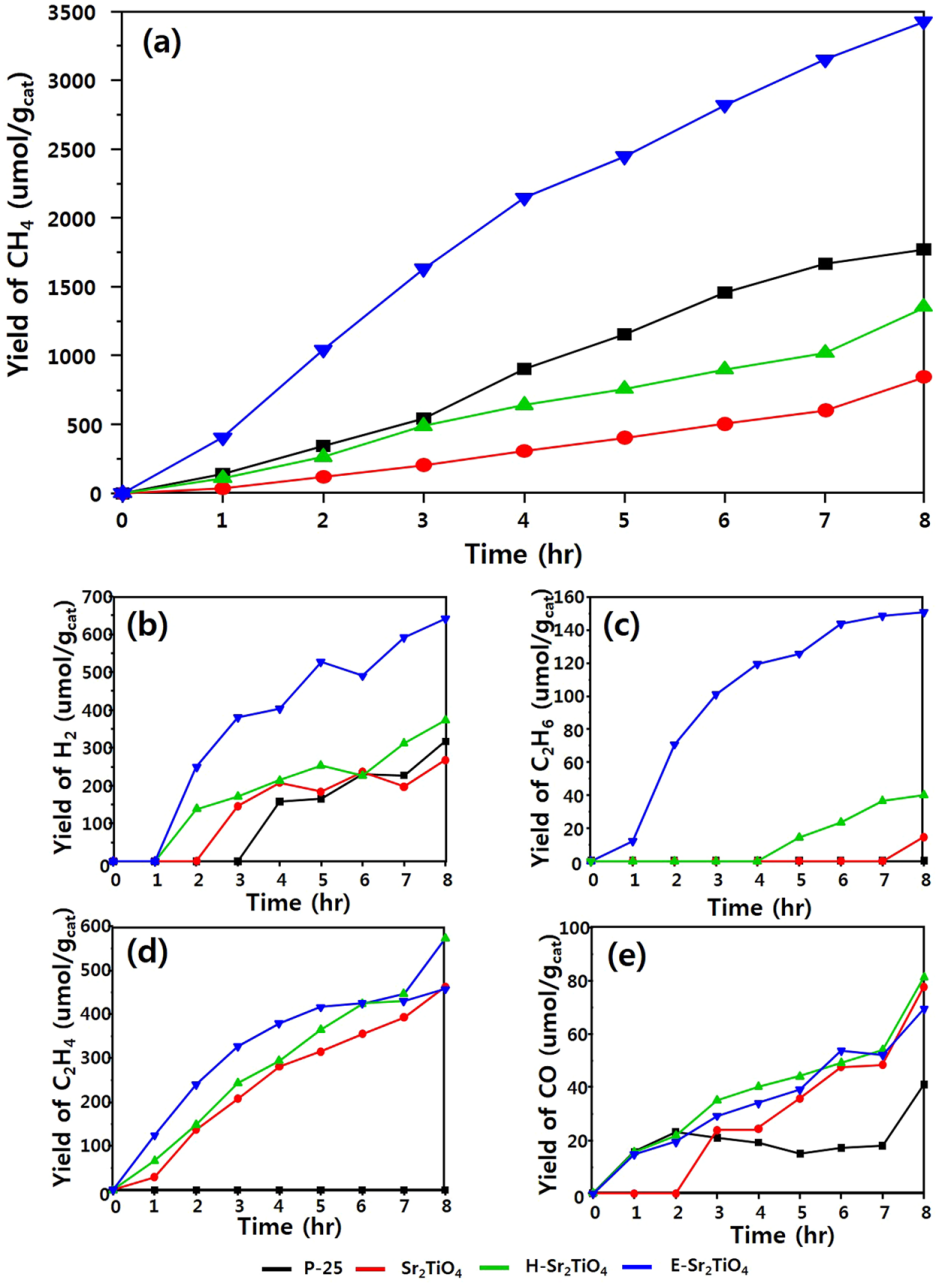
Photocatalytic CO_2_ reduction with H_2_O on catalysts. (**a**) yield of CH_4_, (**b**) yield of H_2_ (**c**) yield of C_2_H_6_ (**d**) yield of C_2_H_4_, and (**b**) yield of CO.

**Figure 13:**
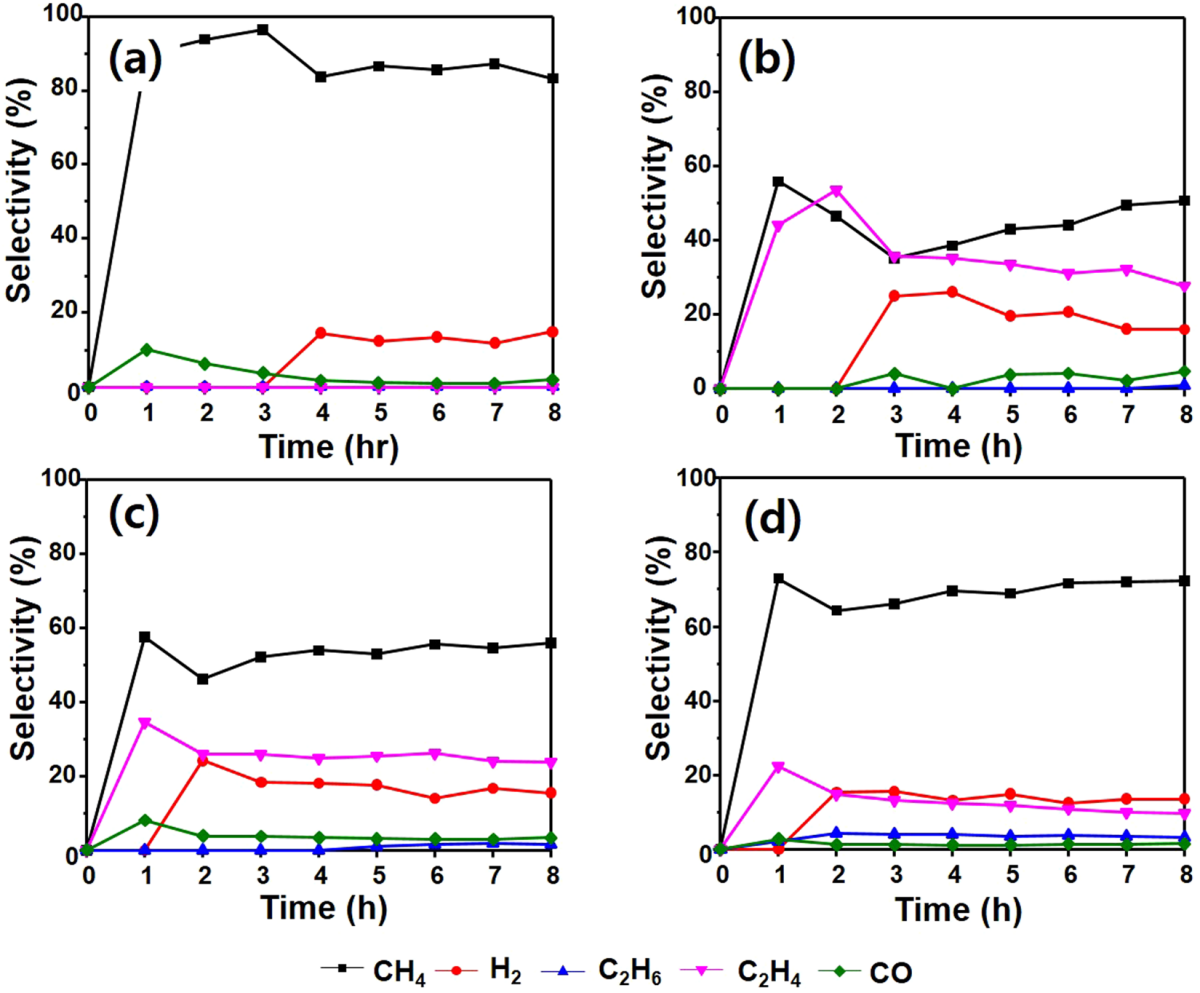
Product distribution on catalysts. (**a**) P-25, (**b**) Sr_2_TiO_4_, (**c**) H-Sr_2_TiO_4_ and (**d**) E-Sr_2_TiO_4._

The products obtained though CO_2_ reduction using the catalysts synthesized in this study are CH_4_, H_2_, C_2_H_6_, C_2_H_4_, and CO. Figure 12 shows the accumulation of the products according to irradiation time. The main product is CH_4_ and the product amounts follow the order CH_4_ > C_2_H_6_ > H_2_ > C_2_H_4_ > CO. Overall, the reactivity of the surface-treated H-Sr_2_TiO_4_ and E-Sr_2_TiO_4_ is better than that of Sr_2_TiO_4_. After 8 h reaction, the rates of CH_4_ production over Sr_2_TiO_4_, H-Sr_2_TiO_4_, and E-Sr_2_TiO_4_ are 844.94, 1353.46, and 3431.77 μmol/g_cat_, respectively. In particular, E-Sr_2_TiO_4_ shows excellent reactivity and produces more CH_4_ than P-25 catalyst, and. Figure 12b shows the amounts of H_2_ produced. H_2_ is a necessary substance for reducing CO_2_. Therefore, the greater the amount of H_2_ generated, the easier the CO_2_ reduction. The amounts of H_2_ generated over Sr_2_TiO_4_, H-Sr_2_TiO_4_, and E-Sr_2_TiO_4_ are 267.73, 373.65, and 640.71 μmol/g_cat_, respectively. Therefore, the CO_2_ reduction reaction is promoted over E-Sr_2_TiO_4_ is higher than the other catalysts. Furthermore, the C_2_H_6_ production over E-Sr_2_TiO_4_ is higher than that over the other catalysts. When E-Sr_2_TiO_4_ is used, the amount of C_2_H_6_ produced after 8 h is 150.8 μmol/g_cat_. If the C_2_H_6_ reacts with H_2_ on the catalysts surface it can be further converted to CH_4_. The amounts of C_2_H_4_ and CO produced over the catalysts are similar. After 8 h, the production of C_2_H_4_ and CO is 457.63–573.33 and 69.35–81.30 μmol/g_cat_, respectively. Figure 13 shows the product distribution on the catalysts. P-25 showed the highest CH_4_ selectivity, which was 80 to 90%. Next, when E-Sr_2_TiO_4_ was used, the CH_4_ selectivity was high and its value was about 70%. Table 3 shows the quantum yield of the catalysts and the overall quantum yield was in the order of E-Sr_2_TiO_4_, P-25, H-Sr_2_TiO_4_, and Sr_2_TiO_4_, which were 2.83, 1.96, 2.93, and 6.20%, respectively. The quantum yield for CO_2_ photoreduction to produce CH_4_ of P-25, Sr_2_TiO_4_, H-Sr_2_TiO_4_ and E-Sr_2_TiO_4_ were 2.69, 1.28, 2.56, 5.21%, respectively. The quantum yield for other products was less than 1% for all catalysts.

**Table 3:** The apparent quantum yield of catalysts.

Catalysts	Quantum yield (%)					
	CH_4_	H_2_	C_2_H_6_	C_2_H_4_	CO	Total
P-25	2.693	0.120	0.000	0.000	0.016	2.829
Sr_2_TiO_4_	1.283	0.102	0.019	0.527	0.030	1.961
H-Sr_2_TiO_4_	2.055	0.142	0.053	0.653	0.031	2.934
E-Sr_2_TiO_4_	5.211	0.243	0.200	0.521	0.026	6.201

**Figure 14:**
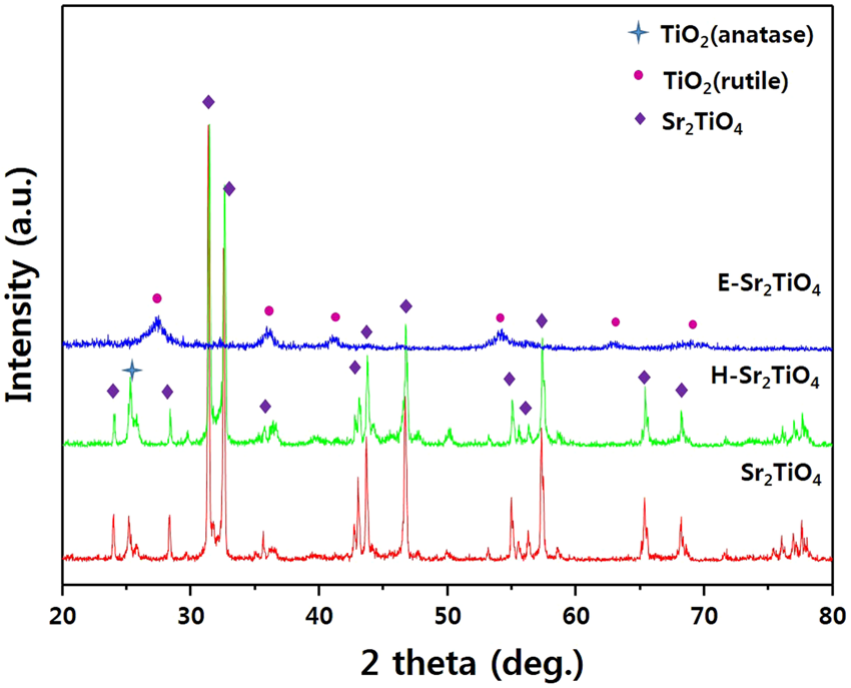
XRD patterns of the Sr_2_TiO_4_, H-Sr_2_TiO_4_, and E-Sr_2_TiO_4_ after the reaction.

The XRD analysis of the catalysts after the reaction was carried out to confirm the structural stability and displayed in Fig. 14. From the XRD analysis results, it was observed that the catalysts structure was remained stable before and after the reaction. Therefore, the catalysts structure was stable during the reaction conditions.

**Figure 15:**
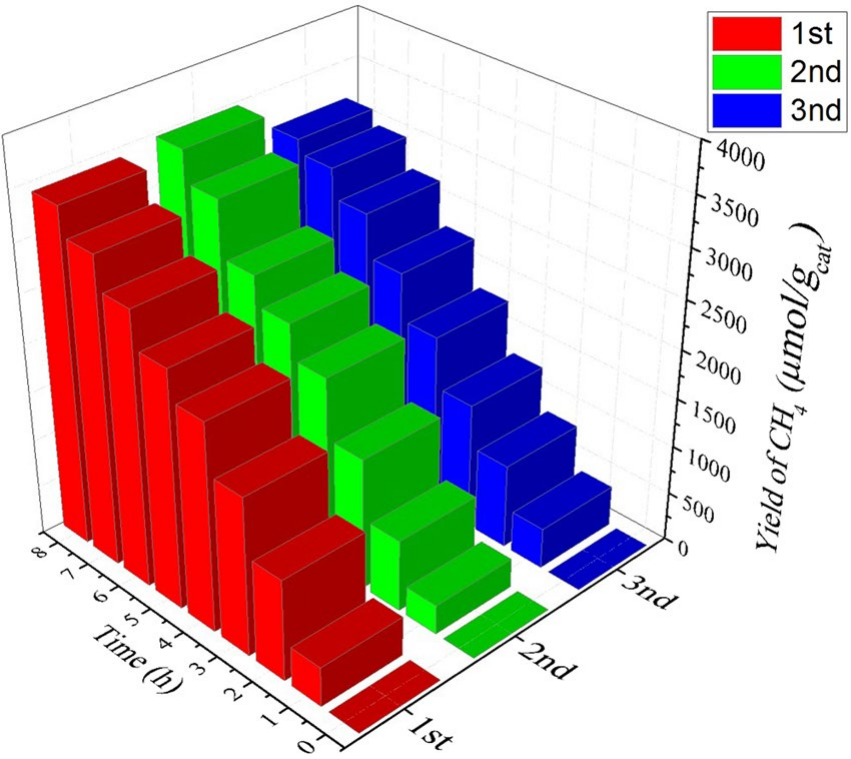
Reusability test of E-Sr_2_TiO_4_ to convert from CO_2_ to CH_4_ using photoreduction.

The photoreaction of E-Sr_2_TiO_4_ showing the best activity was repeated three times. The results for CH_4_ production, the main product, are shown in Fig. 15. A slight amount of difference was observed in the results but similar performance was maintained without deactivation in all three times. Therefore, it was confirmed that the E-Sr_2_TiO_4_ was excellent in not only structural stability but also reusability during the reaction

**Figure 16:**
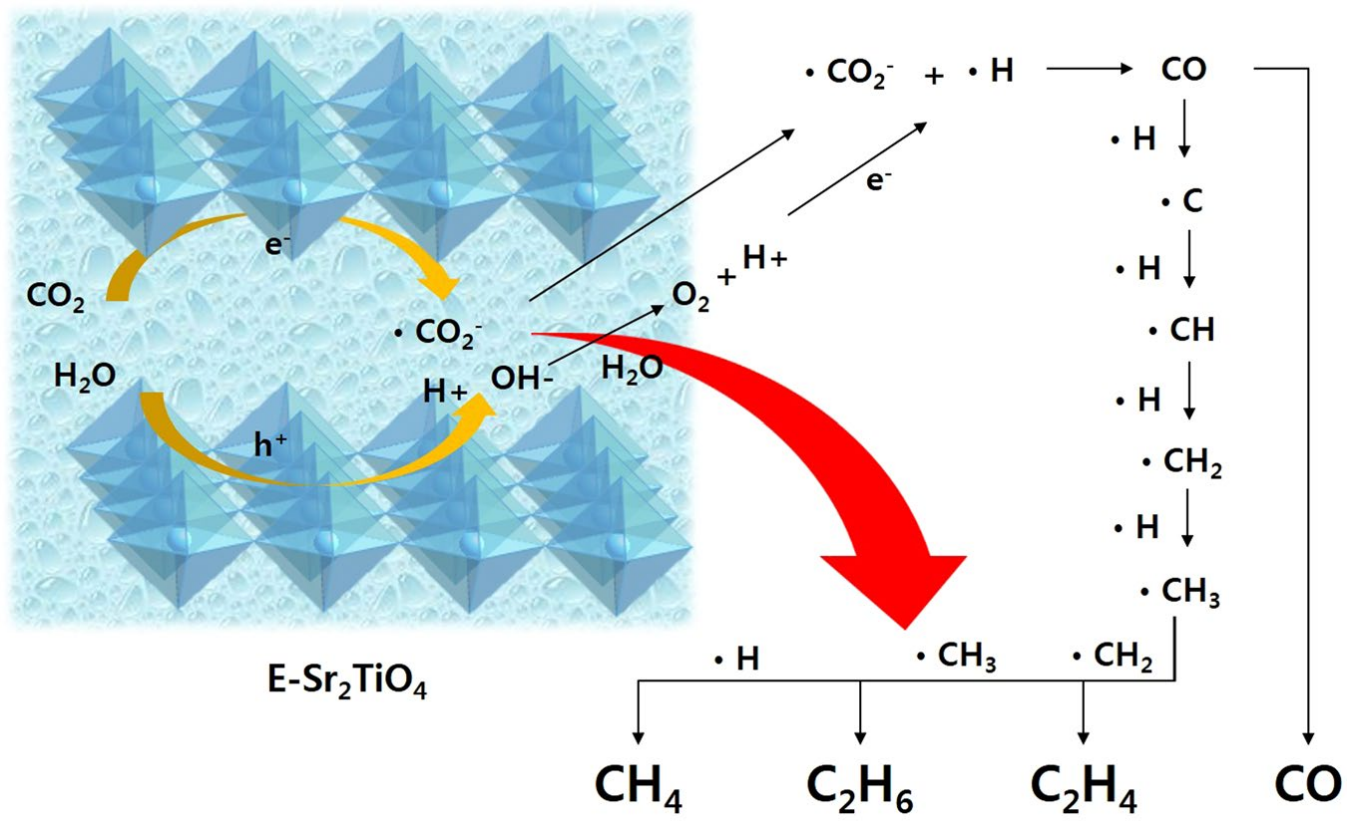
The proposed mechanism for the photo-reduction of CO_2_ with H_2_O on E-Sr_2_TiO_4_.

Based on these results, a plausible reaction pathway over E-Sr_2_TiO_4_, which has the best performance is proposed in Fig. 16. The excited electrons on the exfoliated improved catalyst surface react with CO_2_ to produce ∙CO_2_^−^ radicals, and the holes react with H_2_O to produce OH^−^ and H^+^. Hydrogen radicals (∙H) are formed by the reaction of H^+^ with excited electrons, and then CO is produced by the reaction of ∙H and ∙CO_2_^−^ radicals. CH_4_, C_2_H_6_, and C_2_H_4_ are produced finally as the CO and ∙H radicals continuously react.

## Conclusion

In this study, nanosized layered perovskite Sr_2_TiO_4_ photocatalyst was successfully synthesized by using sol-gel technique with the assistance of citric acid. The surface of layered perovskite Sr_2_TiO_4_ photocatalyst was treated to improve the CO_2_ photoreduction activity. The particles were treated with HNO_3_ to remove the Sr ions present between the layers, and the layers were exfoliated by treatment with TPAOH. The catalyst, E-Sr_2_TiO_4_ showed the rutile TiO_2_ structure after exfoliation because the Sr_2_TiO_4_ structure was collapsed. The shape of the exfoliated thin film was confirmed by TEM. In comparison to Sr_2_TiO_4_ and H-Sr_2_TiO_4_ photocatalysts, the exfoliated catalyst E-Sr_2_TiO_4_ showed an excellent performance in CO_2_ photoreduction to CH_4_, and after 8 h, 3431.77 μmol/g_cat_ of CH_4_ was generated. The reason for the excellent performance of E-Sr_2_TiO_4_ can be explained by the following factors.

First, it has a narrow band gap compared to the other two catalysts, and exhibits reduced electron-hole recombination. Therefore, a relatively larger number of electrons and holes transferred to CO_2_ and H_2_O. Next, a large amount of CO_2_ and H_2_O can interact with the active sites on the surface because it has a large surface area and is well dispersed in the solution. Based on the excellent physical and photochemical properties of the exfoliated layered perovskite catalyst, it may be employed for different photocatalytic applications as well as the CO_2_ photoreduction reactions.

## Methods

### Synthesis of photocatlysts

**Figure 17:**
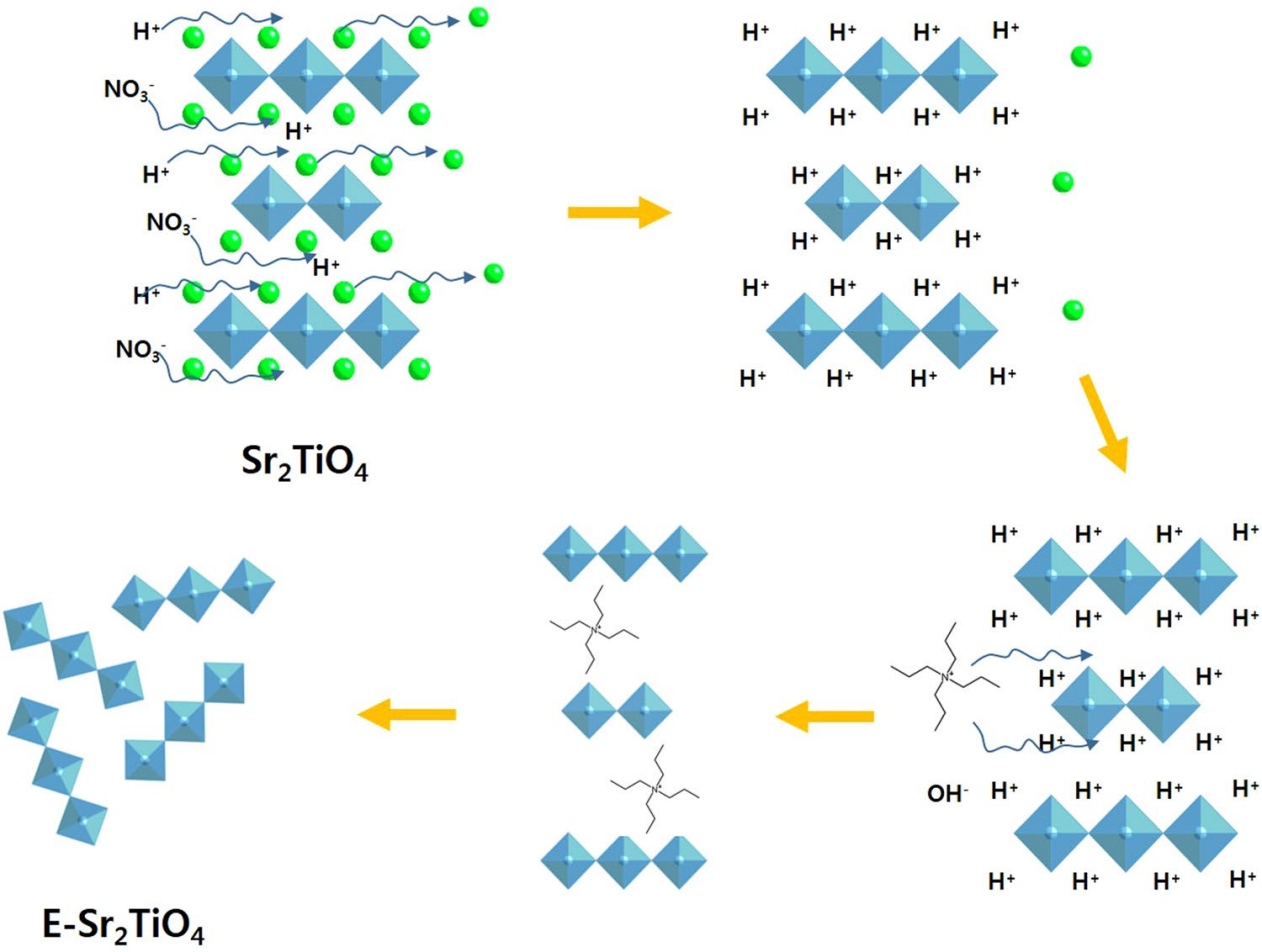
Preparation of Sr_2_TiO_4_, Exfoliation Sr_2_TiO_4_ and H-Sr_2_TiO_4_ catalysts.

The synthesis of layered perovskite Sr_2_TiO_4_ was performed as follows: Strontium nitrate (Sr(NO_3_)_2_, 97.0%, Junsei Chemical, Japan) and titanium isopropoxide (Ti(OCH(CH_3_)_2_)_4_, TTIP, 98.0%, Junsei Chemical, Japan) were used as precursors. First, 0.1 mol of Sr(NO_3_)_2_ was dissolved in double distilled water (100 mL) with continuous stirring. Then, 10 mL of HNO_3_ (60%, OCI company Ltd., Republic of Korea) was added with stirring to prevent hydrolysis. This solution was labeled A. In a separate vessel, 0.05 mol of TTIP was dissolved in EtOH (99.9%, OCI company Ltd., Republic of Korea), and then glacial acetic acid (CH_3_COOH, 99.0%, Ducsan, Republic of Korea) was added with stirring to prevent hydrolysis. This solution was labeled as B. The solutions A and B were then mixed with stirring, and citric acid monohydrate (C_6_H_8_O_7_·H_2_O, 99.5%, Daejung Chemicals and Metals Co Ltd., Republic of Korea), which is a complexing agent for the gel, was added, and the mixture was stirred until it became homogeneous. Then, the solvent was removed without the temperature exceeding 323.15 K to obtain a sol gel, which was subsequently pretreated at 493.15 K. Finally, a white powder was obtained by thermal treatment at 1323.15 K for 6 h. In some cases, hydrogen treatment was then performed at 1123.15 K for 3 h in H_2_ atmosphere. In other cases, exfoliation was achieved by a three-step process, as shown in Fig. 17. Ion exchange of Sr for H cations was carried out in 1 M HNO_3_ for 5 days using ultrasonication. The powder obtained was treated in tetrapropylammonium hydroxide (TPAOH, 25.0%, in water, ACROS, Belgium) for 3 weeks using ultrasonication. The final white precipitate was washed several times with distilled water and ethanol and dried at 343 K for 24 h. The hydrogen-treated and exfoliated Sr_2_TiO_4_ samples were labeled H-Sr_2_TiO_4_ and E-Sr_2_TiO_4_, respectively.

### Characterization of photocatalysts

The structures and crystallinities of the as-prepared Sr_2_TiO_4_, H-Sr_2_TiO_4_, and E-Sr_2_TiO_4_ samples were confirmed with XRD (model MPD from PANalytical) using nickel-filtered CuKα radiation (40.0 kV, 30.0 mA). The morphologies were investigated using HR-TEM (Tecnal G2 F20 S-TWIN, FEI, Netherlands) operated at 200 kV. The presence of different elements was confirmed using the elemental mapping and energy dispersive X-ray spectroscopy (EDS) attached to the TEM setup. The specific surface areas (S_BET_) were calculated according to the Brunauer-Emmett-Teller theory using a Belsorp II mini (BEL, Japan Inc.). The UV-Vis absorption spectra were obtained using a SCINCO Neosys-2000 spectrometer fitted with a reflectance sphere. PL profiles were obtained using a SCINCO FluoroMate FS-2 at room temperature using a He-Cd laser source at a wavelength of 325 nm. XPS measurements were performed on a K-alpha (Thermo Scientific, UK) using Al Kα X-rays as the excitation source. The zeta potential of the material was determined by electrophoretic mobility using an electrophoresis measurement apparatus (ELS 8000, Otsuka Electronics, Japan) with a plate sample cell. Electrophoretic light scattering (ELS) determination was performed in reference beam mode with a 670 nm laser light source at a modular frequency of 250 Hz and a scattering angle of 15°. The standard error of the zeta potential, converted from the experimentally determined electrophoretic mobility, was typically <1.5% with 5% error. To measure the zeta potentials, the samples were dispersed in deionized water or bubbling-CO_2_ water at 0.1 wt%. The final zeta potentials were obtained by averaging 2 or 3 measurements.

### Photocatalytic activity measurements

**Figure 18:**
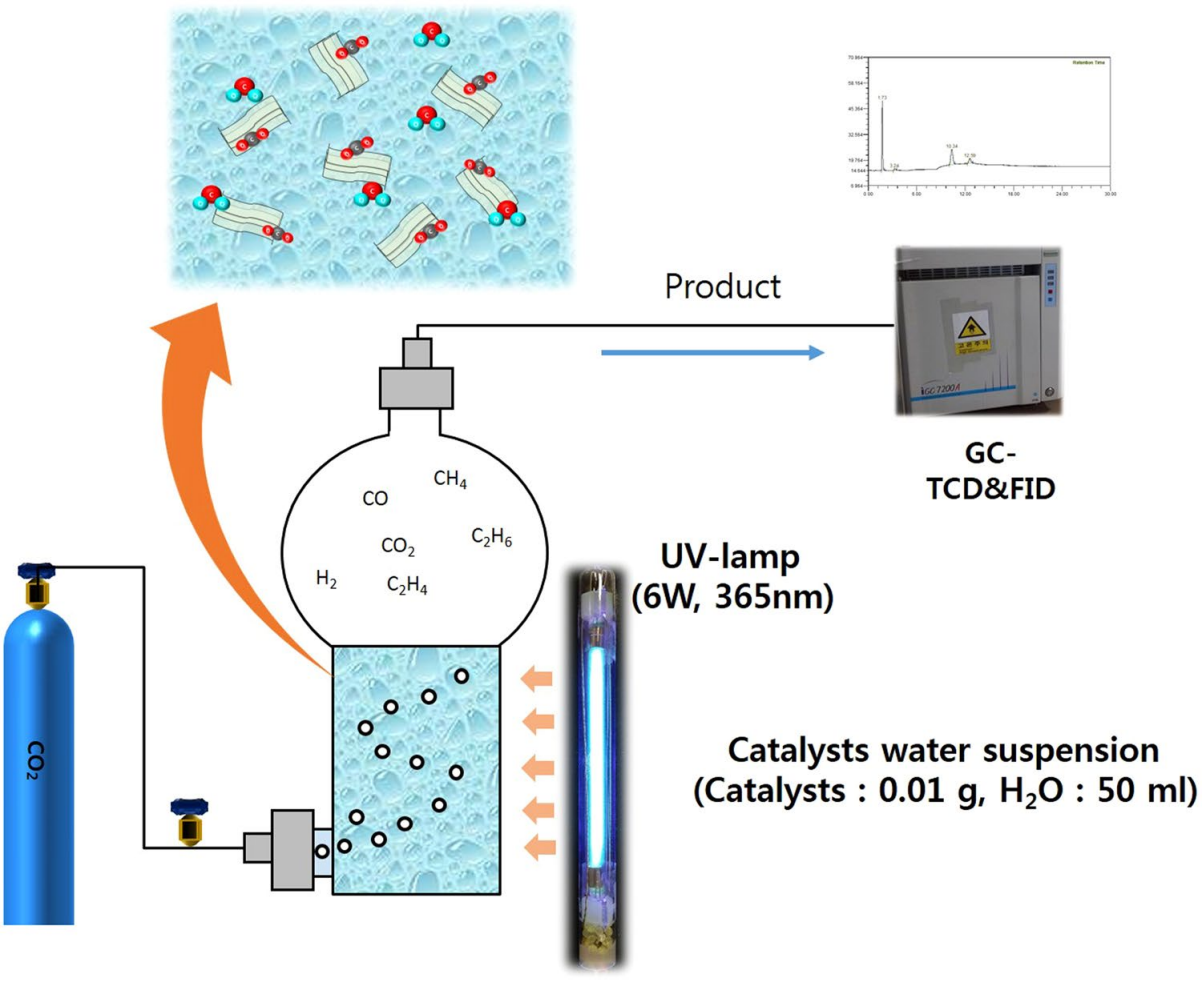
The Schematic diagram of experimental set up of a circulated photo reactor for CO_2_ reduction.

The photocatalytic tests for the reduction of CO_2_ with H_2_O were performed in a photoreactor comprising a quartz chamber with a total volume of 150.0 cm^3^ (Fig. 18). To photoreduce CO_2_, 0.01 g of the catalyst was placed in the reactor chamber with 50 mL of double distilled water, and the reactor was closed. A UV lamp (6 W/cm^2^, 20 cm length × 2.0 cm diameter, Shinan, Republic of Korea) emitting light at 365 nm was used to irradiate the reaction mixture. Supercritical-fluid-grade CO_2_ with a certified maximum hydrocarbon content of <1 ppm was used as the reactant. Before the reaction was initiated by illumination, the reactor was purged with CO_2_ gas for 5 min. The lamp was then switched on to start the experiment. The reaction temperature and pressure were maintained at 303 K and 1 atm, respectively. The gas products were analyzed using a gas chromatograph (iGC7200, DS Science, Republic of Korea) equipped with a thermal conductivity detector (TCD) and a flame ionization detector (FID). The product yield^33^ and quantum yield^63^ during reaction was calculated using following equation (2–3).

2 $${\rm{Product}}\,{\rm{yield}}={\rm{Total}}\,{\rm{of}}\,{\rm{product}}\,({\rm{\mu }}\mathrm{mol})/\mathrm{Amount}\,{\rm{of}}\,{\rm{photocatalyst}}\,{\rm{used}}({{\rm{g}}}_{{\rm{cat}}})$$

3 $${\rm{Quantum}}\,{\rm{yield}}( \% )={\rm{Number}}\,{\rm{of}}\,{\rm{reacted}}\,\mathrm{electrons}/\mathrm{Number}\,{\rm{of}}\,{\rm{incident}}\,{\rm{photons}}\times 100 \% $$
